# Supplementary material for: Single-Cell Analysis Identifies Thymic Maturation Delay in Growth-Restricted Neonatal Mice
Source: Front Immunol. 2018 Nov 1;9:2523. doi: 10.3389/fimmu.2018.02523 (PMC6221967; doi:10.3389/fimmu.2018.02523)
Supplement: Supplementary file 3 [file Data_Sheet_2.docx]

Supplementary Material

Single-Cell Analysis Identifies Thymic Maturation Delay in Growth-Restricted Neonatal Mice

**Wendi A. Bacon^1,2,3*^^, Russell S. Hamilton^2,3^^, Ziyi Yu^4^, Jens Kieckbusch^1,2^, Delia Hawkes^1^, Ada M. Krzak^1^, Chris Abell^4^, Francesco Colucci^1,2^, D. Stephen Charnock-Jones^1, 2*^**

^1^Department of Obstetrics & Gynaecology, University of Cambridge, Cambridge, United Kingdom

^2^Centre for Trophoblast Research, University of Cambridge, Cambridge, United Kingdom

^3^Department of Physiology, Development, & Neuroscience, University of Cambridge, Cambridge, United Kingdom

^4^Department of Chemistry, University of Cambridge, Cambridge, United Kingdom

^^^Co-first Authors

*** Correspondence:**Dr. Wendi Bacon
[wendi.bacon@gmail.com](mailto:wendi.bacon@gmail.com)

Professor D. Stephen Charnock-Jones
[dscj1@cam.ac.uk](mailto:dscj1@cam.ac.uk)

**Dataset S1**

Transcript markers distinguishing each cluster using an adjusted p value < 0.01 are shown here.

T-Cell.SupplementalFiles.zip

Files contained in the zip file. File names correspond to their associated figure in the manuscript

7A - T-Cell.Table.DN.vs.DP.adjp.0.01.csv

7B - T-Cell.Table.DP.vs.TMat.adjp.0.01.csv

7C - T-Cell.Table.WT_DN.vs.P0_DN.adjp.0.01.csv

7D - T-Cell.Table.WT_DP.vs.P0_DP.adjp.0.01.csv

7E - T-Cell.Table.WT_TMat.vs.P0_TMat.adjp.0.01.csv

S7A.01 - T-Cell.Table.1.vs.2.adjp.0.01.csv

S7A.02 - T-Cell.Table.1.vs.3.adjp.0.01.csv

S7A.03 - T-Cell.Table.1.vs.4.adjp.0.01.csv

S7A.04 - T-Cell.Table.1.vs.5.adjp.0.01.csv

S7A.05 - T-Cell.Table.1.vs.6.adjp.0.01.csv

S7A.06 - T-Cell.Table.2.vs.3.adjp.0.01.csv

S7A.07 - T-Cell.Table.2.vs.4.adjp.0.01.csv

S7A.08 - T-Cell.Table.2.vs.5.adjp.0.01.csv

S7A.09 - T-Cell.Table.2.vs.6.adjp.0.01.csv

S7A.10 - T-Cell.Table.3.vs.4.adjp.0.01.csv

S7A.11 - T-Cell.Table.3.vs.5.adjp.0.01.csv

S7A.12 - T-Cell.Table.3.vs.6.adjp.0.01.csv

S7A.13 - T-Cell.Table.4.vs.5.adjp.0.01.csv

S7A.14 - T-Cell.Table.4.vs.6.adjp.0.01.csv

S7A.15 - T-Cell.Table.5.vs.6.adjp.0.01.csv

S8A.1 - T-Cell.Table.WT_1.vs.P0_1.adjp.0.01.csv

S8A.2 - T-Cell.Table.WT_2.vs.P0_2.adjp.0.01.csv

S8A.3 - T-Cell.Table.WT_3.vs.P0_3.adjp.0.01.csv

S8A.4 - T-Cell.Table.WT_4.vs.P0_4.adjp.0.01.csv

S8A.5 - T-Cell.Table.WT_5.vs.P0_5.adjp.0.01.csv

S8A.6 - T-Cell.Table.WT_6.vs.P0_6.adjp.0.01.csv

**Movie S1**

5 - T-Cell.RibosomeMovie.mpg

**Movie S1**: Ribosomal genes identified to be localized on the surface of the ribosome are mapped onto the ribosome structure (red, PDB:6EK0) with a rotation on the y-axis.

**Figure S1.** Representative flow plots from a neonatal thymus. The first row demonstrates how we gated for living, single cells, while the remaining rows show our gating for DN, DP, CD4+ and CD8+ T-cells as listed in detail in Table S2. Samples were processed on a BD LSR Fortessa and the data was analyzed using FlowJo 10.

**Figure S2.** Representative flow plots for innate immune cells from a neonatal spleen. The first row demonstrates how we gated for living, single cells, while the remaining rows show our gating for B-cells and the wide variety of innate immune cells as listed in detail in Table S2. Samples were processed on a BD LSR Fortessa and the data was analyzed using FlowJo 10.

**Figure S3.** Representative flow plots for adaptive immune cells from a neonatal spleen. The first row demonstrates how we gated for living, single cells, while the remaining rows show our gating for various T-cell types as listed in detail in Table S2. Samples were processed on a BD LSR Fortessa and the data was analyzed using FlowJo 10.


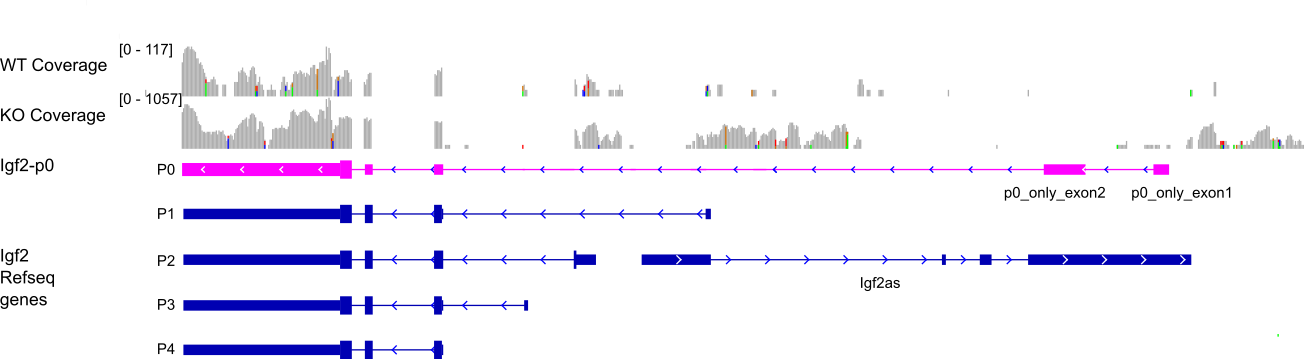


**Figure S4.** P_0_ *Igf-2* transcript not found in thymus. Total reads from both WT and P0 single-cell transcript sequencing were visualized within the *Igf-2* locus. Transcript isoforms from P0 to P4 are shown. All isoforms yield the same translated product.


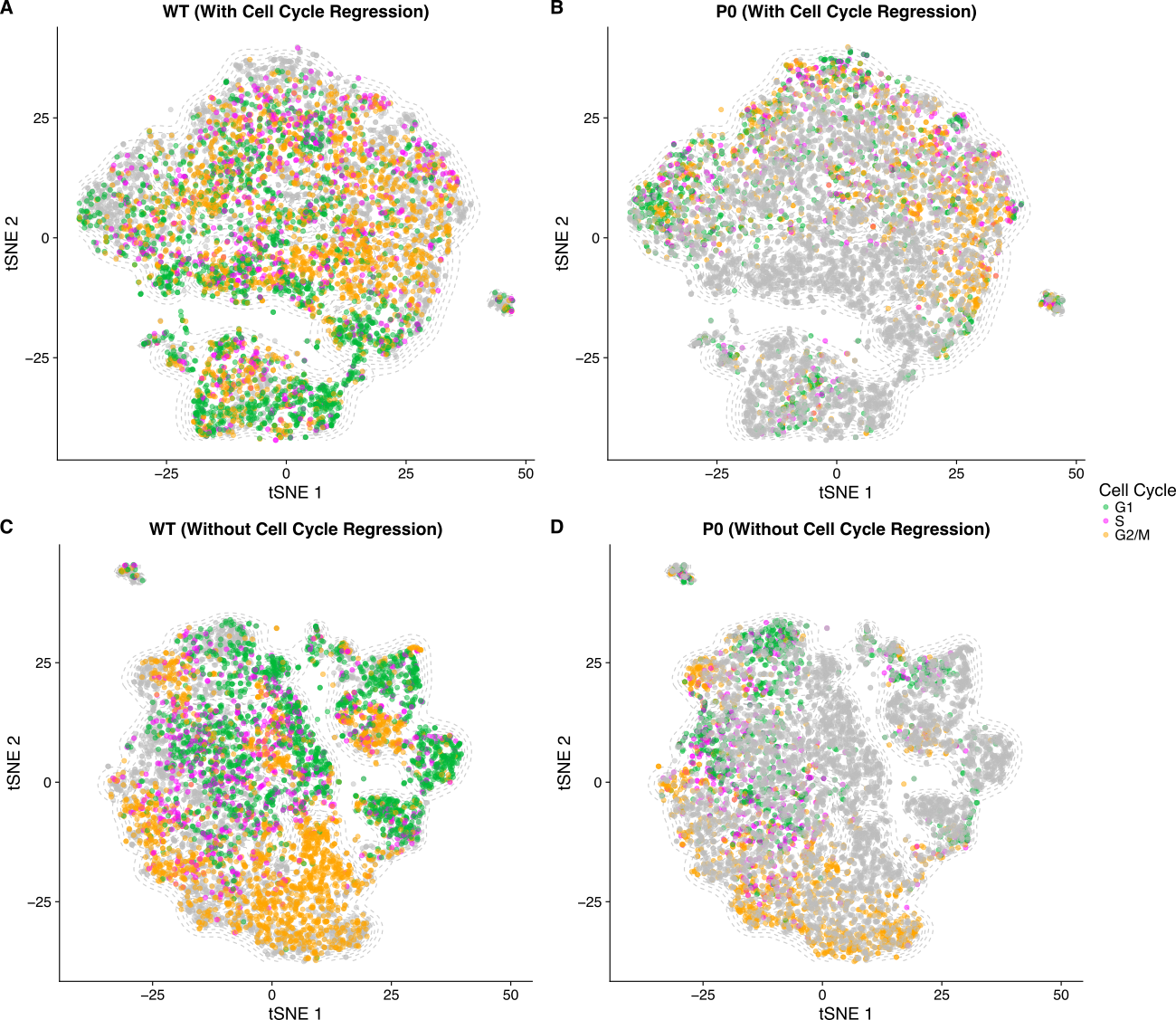


**Figure S5.** Effect of Cell Cycle Regression on Cluster Mapping. Cells were clustered before and after regressing cell cycle determinants. (**A**) WT and (**B**) P0 cells with cell cycle regression, as compared with (**C**) WT and (**D**) P0 cells mapped without cell cycle regression. Cells are colored by genotype (grey if the opposing genotype) and cell cycle phase (G1: Green, S: Pink; G2/M: Orange).


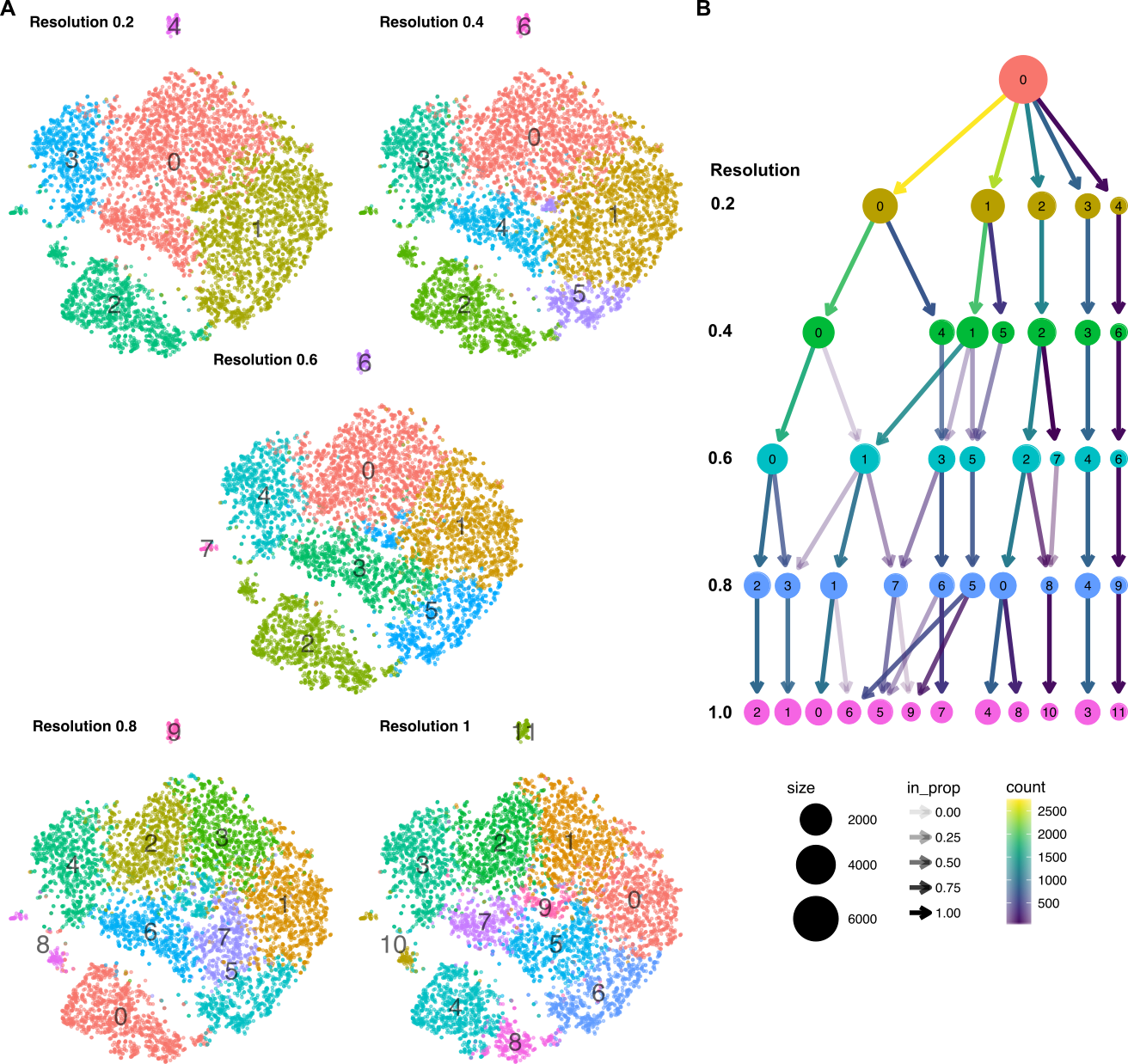


**Figure S6.** Resolution Effects on Cluster Calling. (**A**) In relation to Figure 2, an increase in resolution of cluster calling leads to increased numbers of clusters. (**B***)* The cluster tree diagram shows how consistently clusters are called, for instance the clusters at the right (first row, numbers 3 & 4) are unique clusters no matter what resolution is used. At higher resolutions clusters divide and cells from one or more of the lower resolution clusters contribute.

**
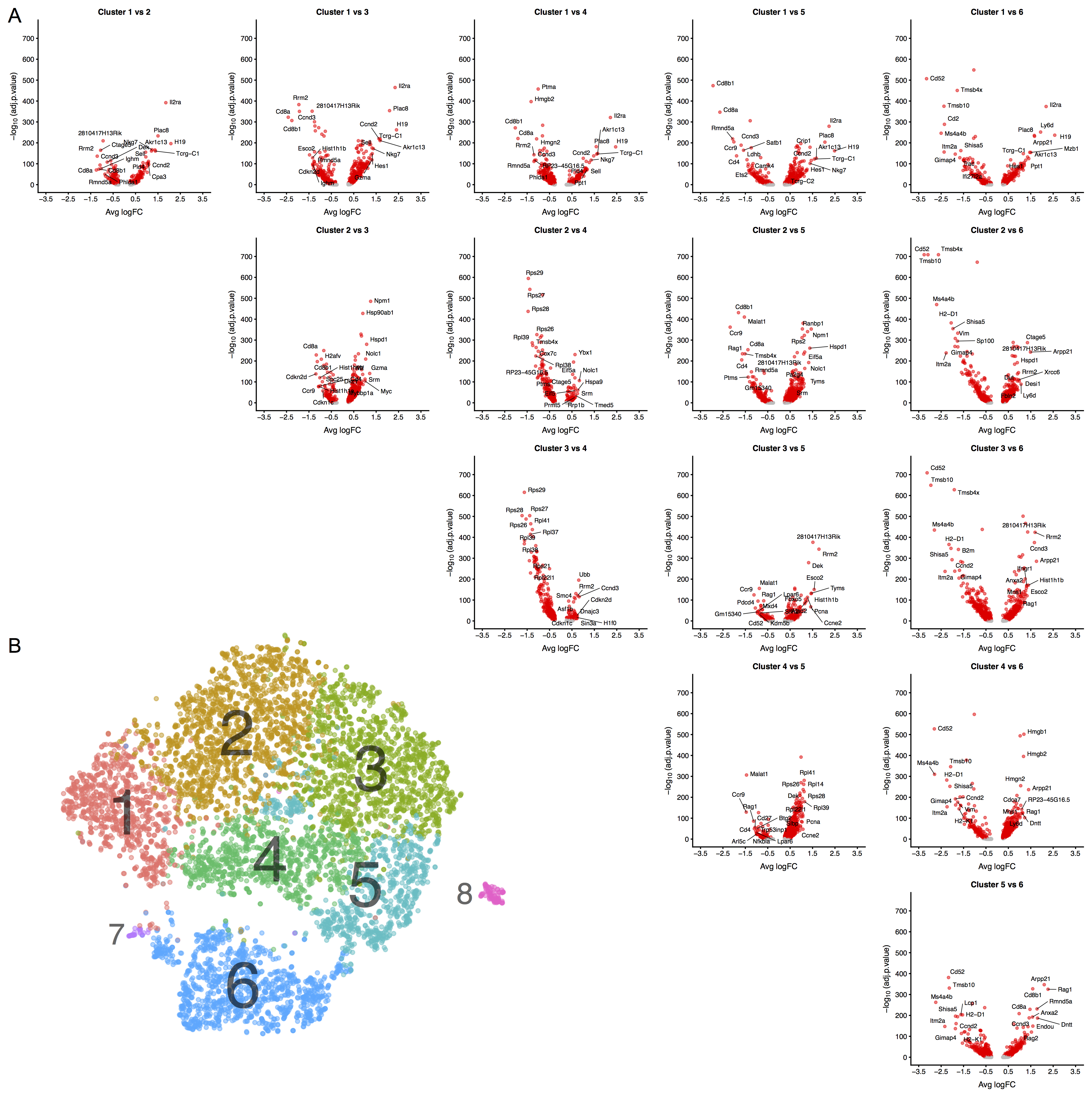
**

**Supplementary Figure 7:** Differential transcript levels between the six largest clusters (1-6). (**A**) Volcano plots for differential transcript levels between each pair of clusters. The identified cluster marker genes were used to assign cell types. The top 10 genes are labelled for +/- average log fold change. (**B**) The clusters compared as shown on the tSNE.


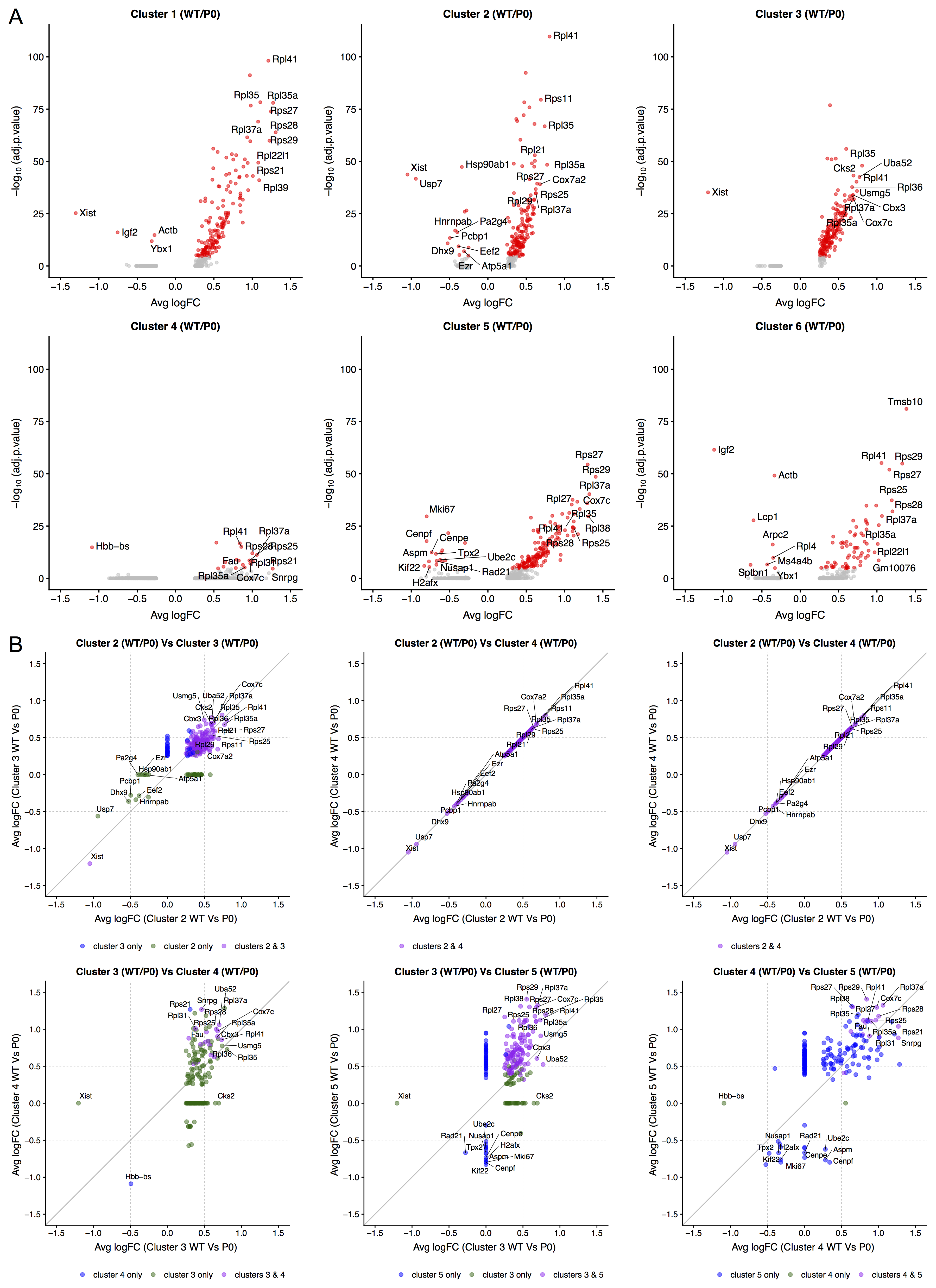


**Supplementary Figure 8:** Differential transcript levels between WT and P0 cells within the six largest clusters (1-6). (**A**) Volcano plots for differential transcript levels for WT vs P0 genotype cells within each cluster revealing the ribosomal protein genes as the dominant set of genes. The top 10 genes are labelled for +/- average log fold change. (**B**) Comparison of the transcript level fold change between DN / DP and DP / T_Mat_ cell type clusters shows a similar enrichment for the ribosomal protein genes (purple).


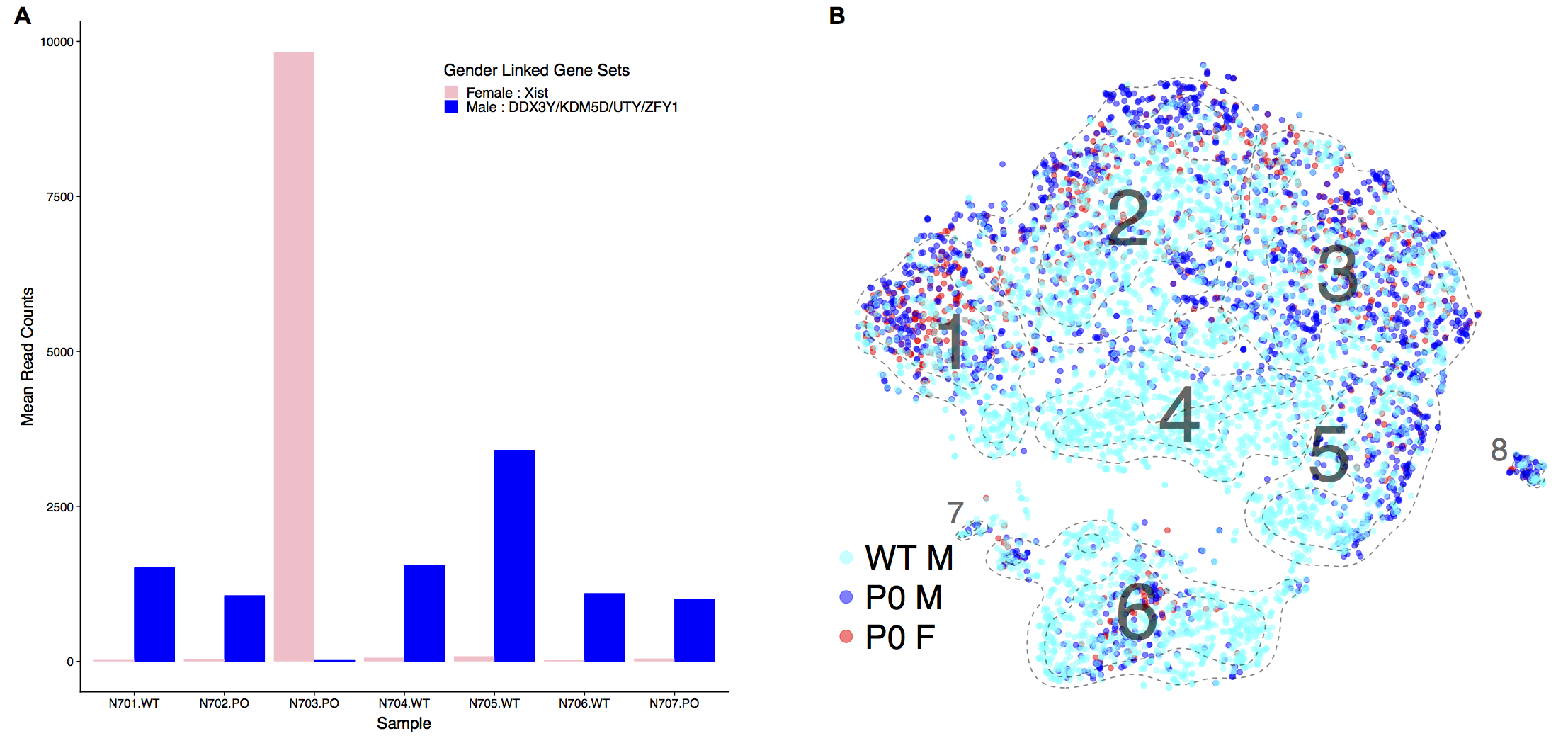


**Supplementary Figure 9:** Cell clustering by sample gender. (**A**) Gender linked gene transcript levels are used to assign the gender to each of the seven samples (4 WT, 3 P_0_). (**B**) Each cell is colored by its genotype and assigned gender in the tSNE revealing no obvious gender bias within clusters.

**Table S1.** Antibodies Used in Flow Cytometry.

| **Marker** | **Clone** | **Company** |
| --- | --- | --- |
| CD11b | M1/70 | BD Biosciences |
| CD11c | N418 | BioLegend |
| CD19 | 1D3 | BD Biosciences |
| CD25 | PC61 | BioLegend |
| CD3 | 17A2, | BioLegend |
| CD4 | RM4-5 | eBioscience |
| CD44 | IM7, | BioLegend |
| CD8a | 53-6.7 | eBioscience |
| Eomes | Dan11mag | eBioscience |
| F4/80 | BM8 | eBioscience |
| FOX3Pm | NRRF-30 | eBioscience |
| Gr1 | RB6-8C5 | BioLegend |
| LyC6 | AL-21 | BD Pharmingen |
| Ly6g | 1A8 | BioLegend |
| MHC-II | M5/114.15.2 | BioLegend |
| mPDCA | eBio129c | eBioscience |
| NK1.1 | PK-136 | BioLegend |
| NKp46 | 29A1.4 | BioLegend |
| SiglecF | E50-2440 | BD Bioscience |
| TCR_β_ | H57-597 | eBioscience |
| TCR_δ_ | GL3 | BioLegend |
| TER-119 | TER-119 | eBioscience |

**Table S2.** Cell marker combinations used to identify splenic and thymic subsets as shown in Figures 1, 4, and 5.

| **Cell Type** | **Identification** |
| --- | --- |
| B cells | CD3^-^ TER119^-^ CD19^+^ |
| ILC1 | CD3^-^ TER119^-^ CD19^-^ NK1.1^+^ NKp46^+^ EOMES^-^ |
| NK Cells | CD3^-^ TER119^-^ CD19^-^ NK1.1^+^ NKp46^+^ EOMES^+^ |
| Conventional Dendritic Cell | CD3^-^ TER119^-^ CD19^-^ NK1.1^-^ MHC^-^II high CD11c high |
| Eosinophils | CD3^-^ TER119^-^ CD19^-^ NK1.1^-^SiglecF^+^ SSC high |
| Monocytes | CD3^-^ TER119^-^ CD19^-^ NK1.1^-^ Ly6G^-^ CD11b^+^ Ly6c^+^ |
| Neutrophils | CD3^-^ TER119^-^ CD19^-^ NK1.1^-^ Ly6G^+^ |
| Plasmacytoid Dendritic Cell | CD3^-^ TER119^-^ CD19^-^ NK1.1^-^ mPDCA^+^ Ly6c^+^ |
| Macrophages | F4/80^+^ |
| T^-^Cell Subtypes | |
| Regulatory | CD19^-^ TER119^-^ CD3^+^ TCRd^-^ TCRβ^+^ CD4^+^ FoxP3^+^ |
| δγ | CD19^-^ TER119^-^ CD3^+^ TCRδ ^+^ |
| αβ | CD19^-^ TER119^-^ CD3^+^ TCRδ^-^ TCRβ^+^ |
| SP CD4^+^ | CD11b^-^ NK1.1^-^ CD19^-^ TER119^-^ CD11c^-^ Gr1^-^ TCRδ ^-^ CD4^+^ CD8^-^ |
| SP CD8^+^ | CD11b^-^ NK1.1^-^ CD19^-^ TER119^-^ CD11c^-^ Gr1^-^ TCRδ^-^ CD4^-^ CD8^+^ |
| DN | CD11b^-^ NK1.1^-^ CD19^-^ TER119^-^ CD11c^-^ Gr1^-^ TCRδ^-^ CD3low/negative CD4^-^ CD8^-^ |
| DP | CD11b^-^ NK1.1^-^ CD19^-^ TER119^-^ CD11c^-^ Gr1^-^ TCRδ^-^ CD4^+^ CD8^+^ |
| DN1 | CD11b^-^ NK1.1^-^ CD19^-^ TER119^-^ CD11c^-^ Gr1^-^ TCRδ^-^ CD3low/negative CD4^-^ CD8^-^CD25low CD44high |
| DN2 | CD11b^-^ NK1.1^-^ CD19^-^ TER119^-^ CD11c^-^ Gr1^-^ TCRδ^-^ CD3low/negative CD4^-^ CD8^-^CD25high CD44high |
| DN3 | CD11b^-^ NK1.1^-^ CD19^-^ TER119^-^ CD11c^-^ Gr1^-^ TCRδ^-^ CD3low/negative CD4^-^ CD8^-^CD25high CD44low |
| DN4 | CD11b^-^ NK1.1^-^ CD19^-^ TER119^-^ CD11c^-^ Gr1^-^ TCRδ^-^ CD3low/negative CD4^-^ CD8^-^ CD25low CD44low |

Table S3. Sequencing and processing statistics for single-cell sequencing.
